# Coil embolization of a bronchial artery aneurysm originating from the false lumen prior complex endovascular repair for type II postdissection thoracoabdominal aneurysm

**DOI:** 10.1016/j.jvscit.2026.102177

**Published:** 2026-02-04

**Authors:** Konstantinos Kotopoulos, Vaiva Dabravolskaite, Cristina Anghel, Konstantinos Koumarelas, Drosos Kotelis, Vladimir Makaloski

**Affiliations:** Department of Vascular Surgery, Inselspital, Bern University Hospital, University of Bern, Bern, Switzerland

**Keywords:** Bronchial artery aneurysm, BrAA, Coil embolization, Chronic aortic dissection, FET, TEVAR

## Abstract

Bronchial artery aneurysm (BrAA) is a rare but potentially life-threatening condition, often associated with aortic pathology. We present a case of a 59-year-old man with a history of Stanford type A acute aortic dissection and subsequent aortic repairs. Surveillance imaging revealed a progressively enlarging right-sided BrAA originating from the false lumen. A staged approach was adopted, beginning with endovascular coil embolization using 18 detachable coils, followed by an open aortic repair. The procedure was successful, with complete aneurysm exclusion and no perioperative complications. This case underscores the technical challenges of BrAA embolization and highlights the efficacy of a multidisciplinary, staged treatment strategy.

Bronchial artery aneurysm (BrAA) is a rare vascular anomaly found in <1% of bronchial angiographies.^1^ Although often asymptomatic, rupture may result in fatal mediastinal hemorrhage. Owing to its potential for life-threatening complications, early detection and intervention are critical.[2], [3], [4] Recent evidence suggests that rupture risk is independent of BrAA size.^4^ In recent years, minimally invasive transarterial embolization has emerged as the preferred management modality with favorable outcomes.[3], [4], [5], [6], [7] This case presents a complex BrAA in a patient with Morbus aneurysmaticus managed with staged coil embolization, highlighting the efficacy of this approach in managing this rare and potentially fatal condition. Informed consent has been obtained for publication of the case report and the accompanying images.

## Case report

A 59-year-old man with a history of extensive aortic disease was referred to our tertiary vascular center for evaluation of an incidentally discovered BrAA. His vascular history included a Stanford type A acute aortic dissection in 2008, for which he underwent emergency ascending aorta and aortic root replacement with a mechanical composite graft and hemiarch repair. Eight years later, the patient underwent open repair of an infrarenal abdominal aortic aneurysm using a bifurcated Dacron graft. He was enrolled in lifelong imaging surveillance owing to chronic dissection with aneurysmal degeneration of the thoracic aorta.

During routine annual follow-up, computed tomography (CT) angiography revealed progressive enlargement of the aortic arch and descending thoracic aorta, with a maximal diameter of 55 mm and requiring a type II postdissection thoracoabdominal repair. Additionally, a 29 × 28 × 30 mm right-sided BrAA was identified. Notably, the BrAA originated from the false lumen of the dissected aorta, adjacent to the T5-T6 vertebral levels (Supplementary Video 1, online only). There were no symptoms attributable to the BrAA, such as chest pain or hemoptysis. Given its growth, morphology, and false lumen origin, the aneurysm was deemed at high risk of rupture despite being asymptomatic.

A multidisciplinary aortic board consisting of cardiac surgeons, vascular surgeons, and radiologist decided on a staged treatment strategy. Owing to the patient's age, good general condition, and higher risk for spinal cord ischemia, we opted for a four-stage treatment. The first stage comprised transarterial coil embolization to exclude the BrAA from the systemic circulation, reducing the risk of rupture during the open arch repair. Next, open arch repair was undertaken using a frozen elephant trunk (FET) technique. Them, he underwent endovascular treatment with a thoracic stent graft (thoracic endovascular aneurysm repair [TEVAR]) and finally, underwent completion with 4× a fenestrated EVAR (FEVAR) for the thoracoabdominal aneurysm.

Under local anesthesia, percutaneous right common femoral artery access was obtained. A 6F Destination sheath (Terumo) was advanced into the thoracic aorta, followed by selective catheterization using a 5.0F Judkins catheter and a 2.4F Progreat microcatheter (Terumo) (Supplementary Video 2, online only). Eighteen Concerto detachable coils (Medtronic) were successfully deployed into the aneurysmal sac and the bronchial artery. Completion angiography confirmed complete exclusion of the BrAA (Supplementary Video 3, online only). The patient recovered uneventfully and was discharged the next day. One month later, the patient underwent the planned reoperation involving a beating-heart FET procedure using a Thoraflex Hybrid Plexus prosthesis (24 × 26 mm) to replace the aortic arch, thus creating a good landing zone for the thoracoabdominal endovascular repair. Six months later, TEVAR was performed to extend the FET as the initial stage of endovascular aneurysm treatment. The completion of the thoracoabdominal aneurysm repair with a 4× FEVAR is planned at 4 months after TEVAR. CT angiography before FEVAR revealed complete exclusion of the BrAA (Supplementary Video 4, online only).

## Discussion

BrAAs are exceedingly rare vascular anomalies, with a reported prevalence of <1% among patients undergoing bronchial angiography.^8^ Their clinical presentation varies significantly, depending on location and size and ranging from incidental findings to catastrophic rupture. Although most BrAAs are asymptomatic, they carry a risk of rupture leading to mediastinal hemorrhage, hemothorax, or massive hemoptysis, depending on their anatomical position. In mediastinal locations, rupture may mimic aortic dissection with acute chest or back pain and hemodynamic compromise, whereas intrapulmonary BrAAs are more often associated with hemoptysis.^1^,^2^,^9^

The present case illustrates a particularly rare entity, a BrAA arising from the false lumen of a chronic Stanford type A aortic dissection. False lumen perfusion of bronchial arteries has been reported in the literature but remains poorly characterized, and even less frequently has it been implicated in aneurysm formation. False lumen perfusion in chronic aortic dissection creates an abnormal hemodynamic environment that may predispose to aneurysm formation in collateral branches, such as the bronchial arteries. The false lumen often maintains systemic or near-systemic pressures, particularly when re-entry tears allow continued inflow, resulting in persistent flow turbulence and wall shear stress.^10^ Over time, these abnormal forces can cause progressive dilation and weakening of the vessel wall. In addition, chronic dissection is associated with vasa vasorum hypertrophy, inflammatory remodeling, and neovascularization, which further compromise vascular integrity and promote aneurysmal degeneration.[11], [12], [13] When bronchial branches originate from or communicate with the false lumen, this combination of high-pressure flow and altered wall biology provides a plausible mechanism for the development of BrAAs. This vascular configuration added substantial technical complexity to the management strategy. The presence of concurrent thoracic aortic aneurysmal degeneration and a history of multiple aortic surgeries further underscored the need for precise and coordinated planning.

Accessing the bronchial artery originating from the false lumen required careful device planning and navigation through tortuous vasculature. The use of a 6.0F sheath for stable proximal access, in combination with a 5.0F leading catheter and a 2.4F microcatheter, allowed for deep coaxial access to the target vessel. This configuration ensured adequate support to deliver embolic material without loss of position, a frequent challenge in such anatomically distorted systems.

The embolization strategy was tailored to minimize the risk of nontarget embolization, especially given the known spinal cord vascularization via bronchial arteries. Among available options (polyvinyl alcohol particles, gelatine sponge, n-butyl cyanoacrylate, and coils) detachable coils were selected owing to their controllability, retrievability before final deployment, and precise localization.[13], [14], [15] Evidence supports that coils are associated with a lower risk of spinal cord ischemia compared with liquid embolic agents,^16^^,^^17^ which is particularly relevant in thoracic aortic pathologies with complex collaterals.

The staged nature of the therapy was critical. Performing endovascular embolization first allowed for controlled exclusion of the aneurysm, which would have posed a high intraoperative bleeding risk during subsequent open surgical repair using the FET technique. This strategy aligns with recommendations for preconditioning or staging high-risk aortic pathologies to mitigate complications such as hemorrhage, spinal cord ischemia, and organ malperfusion.^5^^,^^18^ It also exemplifies the principle of hybrid vascular management, integrating endovascular and open strategies in a stepwise fashion.

From a clinical standpoint, this case highlights the importance of vigilant imaging surveillance in patients with complex aortic pathology. The BrAA in this patient was identified during routine follow-up CT angiography. Without surveillance, the lesion may have remained occult until rupture. Although the literature suggests BrAA rupture is not size dependent,^4^ the expanding morphology and false lumen origin in this case justified intervention.

Notably, few reports have documented BrAAs originating from the false lumen, and even fewer have described successful treatment via coil embolization followed by planned open repair. The closest comparable cases in the literature involve ruptured or ectopic BrAAs treated with stent-assisted embolization or adjunctive endograft placement.^4^^,^^6^ Our case contributes a novel variant to the growing body of evidence supporting safe and effective embolization of BrAAs using minimally invasive methods, even in highly complex anatomical settings.

Complications after bronchial artery embolization are typically minor and include chest pain, postembolization syndrome, and access site hematomas.^19^ Major complications such as spinal cord ischemia, transient ischemic attack, or cortical blindness are rare but serious. Risks are particularly heightened when bronchopulmonary or spinal artery collaterals are present. In our case, superselective catheterization and the use of coils minimized the likelihood of nontarget embolization, and no periprocedural complications were observed.

## Conclusions

This case demonstrates that transarterial coil embolization of the BrAA could be a valuable strategy in a challenging anatomy and bronchial artery arising from the chronic dissection-related false lumen. With appropriate imaging, catheter selection, and embolic technique, this approach can safely eliminate rupture risk and facilitate staged repair in complex aortic patients.

## Funding

None.

## Disclosures

D.K. and V.M. report research and educational grants to the institution from Abbott, Artivion, Cook, Medtronic, Terumo Aortic. V.M. works as consultant and proctor for Medtronic, Terumo Aortic, and Artivion.

## Appendix (online only)

Supplementary Video 1 Preoperative computed tomography (CT) angiography revealing a 29 × 28 × 30 mm right-sided bronchial artery aneurysm (BrAA) originating from the false lumen of the aorta, accompanied by progressive aneurysmal dilation of the aortic arch and descending aorta.

Supplementary Video 2 Pre-embolization angiography demonstrating a 29 × 28 × 30 mm large bronchial artery aneurysm (BrAA).

Supplementary Video 3 Postembolization angiography with complete exclusion of the bronchial artery aneurysm (BrAA).

Supplementary Video 4 Postoperative computed tomography (CT) angiography after frozen elephant trunk (FET) procedure, thoracic endovascular aneurysm repair (TEVAR) extension, coil embolization of the bronchial artery and prior fenestrated endovascular aneurysm repair (FEVAR).
